# Physical Exercise as an Effective Antiaging Intervention

**DOI:** 10.1155/2017/7317609

**Published:** 2017-03-29

**Authors:** N. Garatachea, A. Santos-Lozano, D. C. Hughes, A. Gómez-Cabello, I. Ara

**Affiliations:** ^1^GENUD Research Group, University of Zaragoza, Zaragoza, Spain; ^2^Centro de Investigación Biomédica en Red de Fisiopatología de la Obesidad y Nutrición (CIBERObn), Madrid, Spain; ^3^Instituto Agroalimentario de Aragón (IA2), Zaragoza, Spain; ^4^Faculty of Health and Sport Sciences (FCSD), Department of Physiatry and Nursing, University of Zaragoza, Zaragoza, Spain; ^5^European University of Miguel de Cervantes, Valladolid, Spain; ^6^Research Institute of Hospital 12 de Octubre (“i+12”), Madrid, Spain; ^7^Department of Neurobiology, Physiology and Behavior, University of California, Davis, Davis, CA 95616, USA; ^8^Centro Universitario de la Defensa, Zaragoza, Spain; ^9^CIBER of Frailty and Healthy Aging (CIBER FES), Madrid, Spain; ^10^GENUD Toledo Research Group, Universidad de Castilla-La Mancha, Toledo, Spain

Physical exercise has been well demonstrated as an effective antiaging intervention. Although exercise certainly cannot reverse the aging process, it does attenuate many of its deleterious systemic and cellular effects [1]. This special issue contains a set of selected papers that represent the broad spectrum in which physical exercise can contribute to a healthy aging. As documented in this issue an active lifestyle represents a powerful tool that may be described as a polypill to prevent and/or treat many conditions and diseases [2].

The topics discussed herein include muscle fiber and muscle functioning, protein intake and sarcopenia, cognitive analysis, study of the perception of the instructors, and association of different biomarkers with physical performance.

H. Jee and J.-Y. Lim performed in vitro muscular function to understand better the characteristics and function of the human muscle during aging. The authors found that age and sex differences seemingly reflect mechanical properties at both the single muscle fibre level and whole thigh muscle level. Despite that, the sex differences were more remarkable at both levels. Moreover, an interesting observation was that several of the functional properties studied among different muscle groups were more prominent in the whole thigh muscle than in single muscle fibres. These discrepancies between skinned single muscle fibres and whole thigh muscle-function characteristics in young and elderly human subjects deserve further investigation.

The study by R. Kangas and colleagues utilized a cross-sectional study design with a 10-year follow-up to address the association between physical performance and miRs with respect to master athletes and aging. A set of miRs (FasL, miR-21, and miR-146a), which are influenced by inflammation and cellular homeostasis, were associated with a decline in physical performance and altered during aging. These findings provide support towards the use of miRs as biomarkers for understanding both the aging process and physical performance.

The age-dependent loss of muscle mass and function/strength (sarcopenia) is increasingly recognized as a major risk factor for adverse outcomes in elderly, especially in the frailty older people. A. M. Martone et al. revised a synergistic approach against sarcopenia composed of exercise and protein intake summarizing the available evidence on the association among physical inactivity, insufficient intake of energy and protein, and poor muscle health in older adults. A robust evidence on the effectiveness of exercise and nutrition at preventing negative outcomes associated with sarcopenia and physical frailty and offering substantial therapeutic effects are included in this review.

E. P. Howard et al. analyzed 4,620 community residing elders in independent housing sites spread in 24 US states to draw how short-term, everyday lifestyle options, including physical activity (PA), help an older adult to maintain their cognitive independence. Specifically, PA and recreational activities showed a favorable effect on cognitive decline, at least in the short term. Moreover, formal and informal educations seemed to have cognitive protective factors. So, these factors could be targets to promote health and well-being in older people.

S. Falbo and colleagues highlight the impact of group exercise classes on improving gait performance in elderly individuals after a 12-week intervention. Importantly though, the inclusion of both a single and dual task being performed during the physical exercise intervention leads to improvements in cognitive function. This evidence supports the potential for PA towards improving the quality of life in not only elderly individuals but also possible populations where cognitive diseases are evident.

Finally, K. R. Robinson et al. explored the concept of Chair Based Exercise, that is, “a primarily seated, structured, and progressive exercise program that is part of a continuum of exercise for older people, using a chair to provide stability, and is delivered by instructors that are suitably skilled and trained to work with frail older people.” This kind of exercise is prescribed to frail older people who are adults unable to take part in standing exercise program. Also authors assessed the perception of instructors that develop this type of exercises. The chair based exercise could be a suitable option for promoting PA in a special population.
